# One Sample, Many Insights: The Epidemiological and Public Health Value of Multiplex PCR Respiratory Panels Following the End of the COVID-19 Pandemic

**DOI:** 10.3390/microorganisms14040887

**Published:** 2026-04-16

**Authors:** Vanja Kaliterna, Nora Josipa Savičević, Vinko Zoranić, Marta Righi, Duje Rakić, Anamarija Jurčev Savičević

**Affiliations:** 1 Teaching Institute for Public Health of Split-Dalmatia County, Vukovarska 46, 21000 Split, Croatia; 2 Faculty of Health Sciences, University of Split, R. Boskovica 35, 21000 Split, Croatia; 3 School of Medicine, University of Split, Soltanska 2A, 21000 Split, Croatia; 4 University Hospital Split, Spinciceva 1, 21000 Split, Croatia

**Keywords:** molecular diagnostics, targeted diagnosis, multiplex PCR, acute respiratory infection, respiratory pathogens, coinfections, clinical-decision making, post-pandemic, Croatia

## Abstract

Background: Molecular diagnostics may detect several respiratory pathogens simultaneously with rapid turnaround times. The aim of this study was to determine the frequency and distribution of respiratory pathogens among symptomatic outpatients. Methods: All outpatients presented for testing due to suspected acute respiratory infection between 1 January and 31 December 2024 to the Teaching Institute for Public Health of Split-Dalmatia County, Croatia, and multiplex real-time PCRs for 13 respiratory pathogens were included. Results: Out of 15,437 analyzed panels, 8878 (57.5%) were positive. Single-pathogen infections dominated (82.6%), while co-infections were recorded in 17.4% of panels; therefore, a total of 10,546 individual pathogens were detected, which were mostly viruses (87.0%). The following distribution of pathogens was observed: rhinovirus/enterovirus in 38.9% of positive results, influenza A virus in 14.5%, SARS-CoV-2 in 9.5%, parainfluenza virus in 7.9%, respiratory syncytial virus in 7.3%, *Mycoplasma pneumoniae* in 4.9%, *Bordetella pertussis* in 4.6%, human metapneumovirus in 4.2%, adenovirus in 3.4%, *Chlamydia pneumoniae* in 3.4%, influenza B virus in 1.3%, *Bordetella parapertussis* in 0.1% and *Legionella pneumophila* had one positive result. The first trimester of the year had the highest number of positive test panels (47.0%). Conclusions: Our study demonstrates a predominance of viral pathogens across all age groups and seasons, further supporting guideline-based practice and highlighting the importance of confirming bacterial infection before initiating antibiotic therapy. This insight into the post-pandemic circulation of respiratory pathogens may help inform public health strategies, including improved surveillance, anticipation of seasonal outbreaks, and targeted interventions, thereby supporting future pandemic preparedness and mitigation efforts.

## 1. Introduction

Acute respiratory infections are the most common infections worldwide and are associated with substantial morbidity and mortality [1]. Numerous viral and bacterial pathogens can cause respiratory disease, and their clinical presentations frequently overlap [2]. Recent studies have demonstrated that multiple pathogens may coexist in the same patient, challenging traditional diagnostic assumptions and complicating clinical decision-making [3].

Upper respiratory tract infections are generally mild and self-limiting, with symptoms typically lasting seven to ten days. Most are viral in origin, although complications such as otitis media in children and sinusitis in older adults may occur. Common symptoms include nasal congestion, sneezing, sore throat, cough, and, occasionally, fever [4]. Lower respiratory tract infections may present as acute bronchitis, exacerbations of chronic bronchitis, or pneumonia [5].

Distinguishing bacterial from viral infections based solely on clinical features is challenging, frequently leading to unnecessary antibiotic prescriptions and contributing to antimicrobial resistance [6]. Molecular diagnostics have significantly advanced clinical microbiology, particularly multiplex real-time PCR assays that detect several respiratory pathogens simultaneously with rapid turnaround times. Multiplex PCR is considered the most sensitive and specific diagnostic approach for identifying respiratory pathogens and differentiating viral from bacterial infections [7]. Rapid antigen tests may be useful in certain settings but have limited sensitivity [8]. Early and accurate pathogen identification improves patient management, particularly for high-risk groups, and reduces inappropriate antibiotic use.

Treatment of viral respiratory infections is primarily supportive, although antiviral therapy may be indicated for selected pathogens [9,10,11]. Bacterial respiratory infections require antibiotics tailored to the specific organism, with macrolides commonly used for *Bordetella pertussis* [12,13]; macrolides, tetracycline, and quinolones for *Chlamydia pneumoniae*, and *Mycoplasma pneumoniae* [14,15]; and macrolides or quinolones for *Legionella pneumophila*. The age-related differences in quinolone use highlight the need for careful consideration of patient age when selecting antimicrobial treatment [16]. In addition to immunization strategies (e.g., against SARS-CoV-2, influenza, respiratory syncytial virus, and *B. pertussis)*, measures such as regular hand hygiene, covering the mouth and nose during coughing or sneezing, self-isolation when symptomatic, and adequate ventilation of enclosed environments significantly reduce the risk of transmission [17].

The central objective of this study is to test the hypothesis that the post-pandemic epidemiological landscape of respiratory pathogens in the Mediterranean has undergone a shift in seasonality and co-infection complexity. While traditional surveillance often relies on single-pathogen testing, this study utilizes high-resolution epidemiological characterization to demonstrate that large-scale multiplex PCR testing provides a ‘diagnostic mandate’ for clinical decision-making. By analyzing over 15,000 cases, we seek to determine how these shifts—specifically the emergence of off-season peaks and high viral–viral co-infection rates—should redefine regional public health strategies and antimicrobial stewardship in a primary care setting. This insight into the post-pandemic circulation of respiratory pathogens may help inform public health strategies, including improved surveillance, anticipation of seasonal outbreaks, and targeted interventions, thereby supporting future pandemic preparedness and mitigation efforts [18].

## 2. Materials and Methods

This retrospective observational study was conducted at the Department of Clinical Microbiology, Teaching Institute for Public Health of Split-Dalmatia County (TIPH SDC), Split, Croatia, over a one-year period, from 1 January to 31 December 2024.

### 2.1. Participants

The study included all outpatients who, during the study period, were referred by their primary care physicians (general practitioners, pediatricians, and epidemiologists) to TIPH SDC for testing due to suspected acute respiratory infection. Referral for suspected respiratory infection was the sole inclusion criterion for sample collection. The study did not apply any exclusion criteria.

### 2.2. Procedures

Nasopharyngeal swabs were collected by authorized TIPH SDC healthcare personnel and placed into tubes containing transport medium (Hanks). Specimens were then stored at 4 °C and processed within 24 h of collection.

All samples were analyzed using the Respiratory Pathogens B 16-well panel (REF 20612; AusDiagnostics, Mascot, Australia). This multiplex PCR panel simultaneously detects 13 respiratory pathogens, including eight viruses: severe acute respiratory syndrome coronavirus 2 (SARS-CoV-2), influenza A virus, influenza B virus, respiratory syncytial virus, parainfluenza virus, human metapneumovirus, adenovirus, and rhinovirus/enterovirus, and five bacteria: *Chlamydia pneumoniae*, *Mycoplasma pneumoniae*, *Legionella pneumophila*, *Bordetella pertussis*, and *Bordetella parapertussis*.

According to the manufacturer’s instruction for use (RIDA^®^Plex Respiratory Viruses 16 well IFU, Version 20, R-Biopharm AG, Darmstadt, Germany), procedures started with automated nucleic acid extraction that was performed using the Viral DNA and RNA Kit (Tianlong, Xi’an, China) in combination with the GeneRotex 96 extraction system (Tianlong, China). Subsequently, a pipetting robot (Myra, BMS Systems, Upper Coomera, Australia) distributed the extracted nucleic acids and PCR reagents into microplate wells for further analysis using multiplex tandem PCR (MT-PCR) with the Respiratory Pathogens B 16-well panel on the Ultraplex 3 system (AusDiagnostics, Mascot, Australia). It is a semi-automated in vitro diagnostic (IVD) test designed for the identification of respiratory pathogens from appropriate clinical specimens, including nasal, throat, and nasopharyngeal swabs, as well as nasopharyngeal and tracheal aspirates, and bronchial and bronchoalveolar lavage samples. Workflow parameters for nucleic acid extraction and PCR were as follows: 200 µL of sample was used as input for automated nucleic acid extraction, and nucleic acids were eluted in a final volume of 50 µL. For each PCR reaction, 10 µL of the extracted nucleic acid was used. Assays utilize Multiplex Tandem PCR (MT-PCR) technology for the amplification of target DNA and/or RNA, rather than conventional real-time quantitative PCR. MT-PCR consists of two amplification steps. The first step is a primary (non-specific) amplification, during which all targets are simultaneously pre-amplified in 18 amplification cycles. The second step is a secondary (target-specific) amplification, performed in individual wells using nested, target-specific primers, with one reaction per target. Amplification data are analyzed by the AusDiagnostics analysis software (Multiplex-Tandem Assay Setup Software, version 1.12.15, AusDiagnostics Ply Ltd., Mascot, Australia), which automatically evaluates amplification and melt curve data and assigns qualitative results, as detected or not detected. Result interpretation is based on an algorithm that incorporates amplification curve characteristics, melt curve analysis, internal controls, and assay calibration parameters. The typical turnaround time of the multiplex PCR assay is approximately 3 h. Positive and negative controls were included in each analytical run. In addition, an endogenous internal control (a reference human gene) was used to assess sample quality. Sample Adequacy Control failed in <5% of samples; these samples were re-tested from the extraction step. If the Sample Adequacy Control failed again, sample re-collection was recommended. A synthetic internal control (SPIKE) was also included to monitor potential PCR inhibition and to verify instrument performance.

The diagnostic performance of the applied multiplex assays was interpreted based on manufacturer validation data and published studies, demonstrating high diagnostic accuracy, with high sensitivity (>99% for most targets) and specificity (>99%). Overall agreement with reference PCR methods was reported at approximately 98–99% in clinical evaluations [19,20]. The clinical relevance of accurate viral detection and comprehensive respiratory panels was further supported in the literature [20,21].

### 2.3. Statistical Methods

We used the MS Excel application from the Microsoft Office Standard 2013 package for the descriptive statistics. An online chi-square calculator was used to check if there was a difference in the number of positives due to the gender of the tested person [22]. A *p*-value of less than 0.05 was accepted as an indicator of statistical significance.

For the purpose of this study, we divided the tested individuals into the following age groups: <1 (infants), 1–6 (preschool-aged children), 7–14 (elementary school-aged children), 15–18 (adolescents), 18–64 (adults), and ≥65 (older adults).

## 3. Results

### 3.1. Test Panels by Age and Sex

Between 1 January and 31 December 2024, a total of 15,437 multiplex PCR panels for respiratory pathogen detection were performed at the Teaching Institute for Public Health of Split-Dalmatia County in patients presenting with symptoms of acute respiratory infection. Of these, 8483 (55.0%) tests were conducted in female patients and 6954 (45.0%) in male patients (Table 1).

Of the 15,437 panels, 8878 (57.5%) yielded positive results, with a higher number of positive panels recorded in females (4710; 53.05%) than males (4168; 46.95%).

However, when each sex was analyzed separately, 55.5% of tested females (4710/8483) had positive results, compared with 59.9% of tested males (4168/6954). This difference was statistically significant, indicating a higher proportion of positive respiratory test panels among males than females (χ^2^ = 13.605, *p* < 0.001).

Participants ranged in age from less than 1 year to 102 years. The mean age of patients with positive results was 23.4 years, with a mean age of 19.6 years for males and 26.7 years for females. Among all positive panels (N = 8878), the highest proportions were observed in adults aged 19–64 years (2933; 33.3%) and preschool children aged 1–6 years (2718; 30.6%), followed by school children aged 7–14 years (1734; 19.5%). Each of the remaining age groups accounted for less than 10% of positive results.

**Figure 1:**
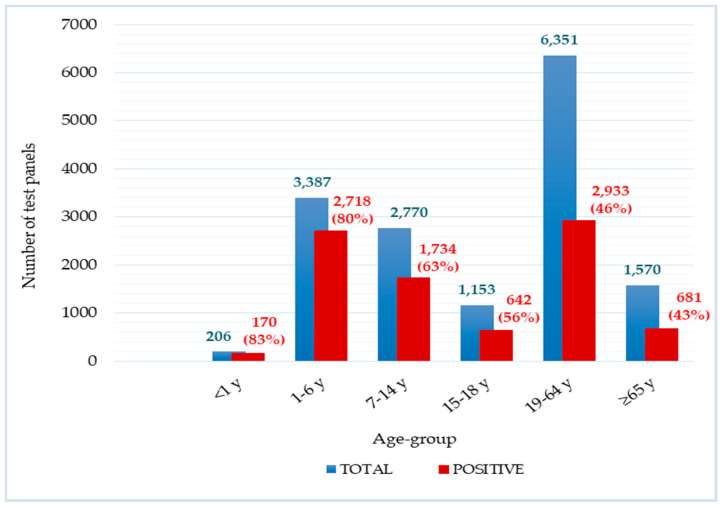
Distribution of participants by age group and test panel results (N = 15,437). Age groups were defined as: <1 year (infants), 1–6 (preschool-aged children), 7–14 (school-aged children), 15–18 (adolescents), 18–64 (adults), and ≥65 (older adults).

However, when analyzing the age distribution among the total of 15,437 tests performed, quite different proportions were observed. Although adults aged 19–64 years accounted for the largest number of tests (N = 6351), only 46.0% (2933) were positive. In contrast, fewer tests were performed in infants (N = 206) and preschool-aged children (N = 3386), but these groups showed high positivity rates of 83.0% (170) and 80.0% (2718), respectively. The distribution of positive test panels by age is shown in Figure 1.

### 3.2. Positive Results by Pathogens and Age Groups

**Figure 2:**
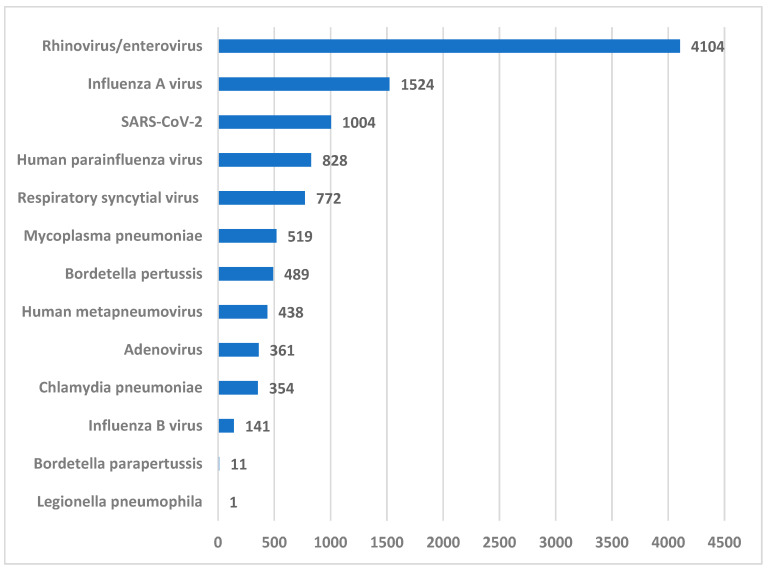
Distribution of positive respiratory test panel results by pathogen (N = 10,546).

Among the 10,546 positive detections, the distribution of pathogens was as follows: rhinovirus/enterovirus in 4104 cases (38.9%), influenza A virus in 1524 (14.5%), SARS-CoV-2 in 1004 (9.5%), parainfluenza virus in 828 (7.9%), respiratory syncytial virus in 772 (7.3%), *Mycoplasma pneumoniae* in 519 (4.9%), *Bordetella pertussis* in 489 (4.6%), human metapneumovirus in 438 (4.2%), adenovirus in 361 (3.4%), *Chlamydia pneumoniae* in 354 (3.4%), and influenza B virus in 141 cases (1.3%), as shown in Figure 2.

The lowest positivity rates were observed for *Bordetella parapertussis* (11 detections; 0.1%) and *Legionella pneumophila*, with one positive result.

The incidence of individual respiratory pathogens also varied across age groups (Table 2). Certain pathogens were more prevalent in younger age groups, others in adults, while some were more frequently detected in older patients.

Among 217 positive results in infants, the most prevalent pathogen was rhinovirus/enterovirus (129; 59.4%), followed by parainfluenza virus (34; 15.7%) and SARS-CoV-2 (22; 10.1%).

Among 3635 positive results in preschool-aged children, rhinovirus/enterovirus dominated (1718; 47.3%), followed by parainfluenza virus (426; 11.7%) and respiratory syncytial virus (396; 10.9%).

Of the 2112 positive detections in the school-aged group, rhinovirus/enterovirus was the most frequently detected (772; 36.6%), followed by influenza A virus (302; 14.3%) and *Mycoplasma pneumoniae* (240; 11.4%).

Among the 726 detected pathogens in adolescents, rhinovirus/enterovirus was the most frequently identified (244; 33.6%), followed by influenza A virus (168; 23.1%).

The most frequent pathogen among 3137 positive adults was also rhinovirus/enterovirus (1039; 33.1%), followed by influenza A virus (710; 22.6%) and SARS-CoV-2 (518; 16.5%).

Among 719 positive results in older adults, SARS-CoV-2 was the most frequently detected pathogen (213; 29.6%), followed by rhinovirus/enterovirus (202; 28.1%) and influenza A virus (114; 15.9%).

Regarding pathogen-specific positive results, rhinovirus/enterovirus was the most frequently detected pathogen in this study, with 4104 positive results. Of these, the highest prevalence was observed in preschool-aged children (1718: 41.9%), followed by adults aged 19–64 years (1039; 25.3%) and school-aged children (772; 18.8%).

Influenza A virus was detected in 1524 test panels, with the highest number of positive results observed in adults (710; 46.6%), followed by school-aged children (302; 19.8%) and preschool children (229;15.0%).

Among participants, SARS-CoV-2 was detected in 1004 cases and was the most common among adults (518; 51.6%) and older adults aged ≥65 years (213; 21.2%).

Parainfluenza virus was detected in 828 test panels, with the highest number of positive results observed in preschool-aged children (426; 51.4%), followed by adults (179; 21.6%).

Among the 772 positive detections of respiratory syncytial virus, more than half occurred in preschool-aged children (396; 51.3%). A substantial proportion of positive results were also recorded in adults (134; 17.4%) and in those aged 7–14 years (132; 17.1%).

*Mycoplasma pneumoniae* was detected in 519 test panels and was most frequently identified in school children aged 7–14 years (240; 46.2%), followed by adults (107; 20.6%) and preschool-aged children (95; 18.3%).

Of the 489 positive *Bordetella pertussis* detections, the highest number occurred in the 7–14 year age group (157; 32.1%), followed by adults (146; 29.9%) and preschool-aged children (112; 22.9%).

Human metapneumovirus was detected in 438 test panels and was most frequently identified in preschool-aged children (168; 38.4%), followed by adults (99; 22.6%) and school-aged children aged 7–14 years (92; 21.0%).

Of the 361 positive adenovirus detections, the majority occurred in preschool children aged 1–6 years (248; 68.7%).

Among the 354 positive *Chlamydia pneumoniae* detections, the highest proportions were observed in school children aged 7–14 years (139; 39.3%), followed by adults (104; 29.4%) and preschool children (83; 23%).

Influenza B virus was detected in 141 test panels, most frequently in adult patients (56; 39.7%), followed by those aged 7–14 years (36; 25.5%) and 1–6 years (30; 21.3%).

*Bordetella parapertussis* was detected in only 11 cases, and it was equally frequent among age groups 1–6 years, 7–14 years, and 19–64 years (each in 3 cases, 27.3%).

### 3.3. Co-Infections

Because some test panels detected two or more respiratory pathogens, a total of 10,546 positive results for individual pathogens were recorded from 8878 positive test panels. Among all detections, viruses predominated (9172; 87%), whereas bacteria accounted for 1374 detections (13%).

Among positive panels (N = 8878), single-pathogen infections were most frequently detected (7337 panels; 82.6%). In nearly one-fifth of the panels, more than one pathogen was isolated (1541 panels; 17.4%). Most commonly, two microorganisms were identified (1414 panels; 16%), while three pathogens were detected in 127 panels (1.4%).

Among the 1414 panels in which two pathogens were detected, the most frequent finding was the presence of two respiratory viruses (1022 panels; 72.3%), whereas combined bacterial–viral infections were observed in 388 panels (27.4%). Bacterial–bacterial co-infections were rare, occurring in only four panels (0.3%). Among dual co-infections, the most common combinations were rhinovirus/enterovirus and parainfluenza virus (in 232 panels), respiratory syncytial virus and rhinovirus/enterovirus (in 159 panels), as well as adenovirus and rhinovirus/enterovirus (in 127 panels). In dual co-infections, rhinovirus/enterovirus was the most frequently detected virus (in 1138 panels), while *Bordetella pertussis* was the most commonly identified bacterium (in 145 panels).

Among triple infections (N = 127), viral–bacterial co-infections were the most common (in 67 panels; 52.76%), while viral–viral co-infections were identified in 60 panels (47.24%). Bacterial–bacterial co-infections were not detected. Among viruses, rhinovirus/enterovirus was the most frequently identified in triple co-infections (in 113 panels), and among bacteria, *Bordetella pertussis* was the most common (in 24 panels). The most frequently observed combinations were adenovirus, rhinovirus/enterovirus, and parainfluenza virus, as well as adenovirus, respiratory syncytial virus, and rhinovirus/enterovirus (10 panels each).

### 3.4. Positive Test Panel Results by Trimesters

**Figure 3:**
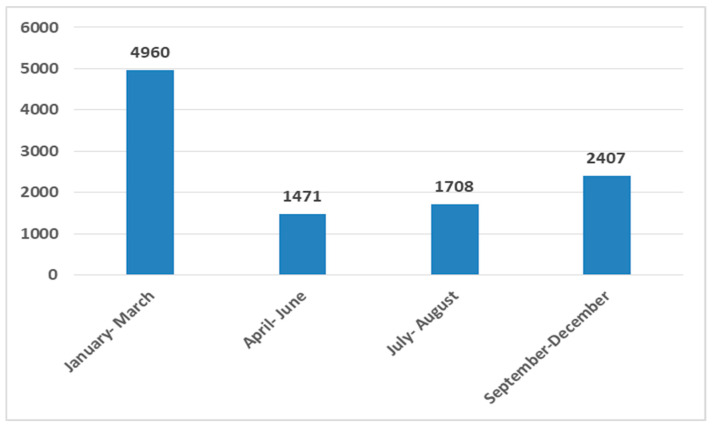
Distribution of positive respiratory pathogen panel results by trimester of the year (N = 10,546).

When pathogen distribution was analyzed by time of year (trimesters), the highest number of positive test panel results was observed in the first trimester (4960), accounting for nearly half of all positive panels (47.0%; 4960/10,546), whereas the lowest number was recorded in the second trimester (14.0%; 1471/10,546) (Figure 3). In the third trimester, 16.2% (1708) of positive cases were detected, while in the fourth trimester, a total of 22.8% (2407) were identified. The distribution of positive test panel results across trimesters showed a statistically significant deviation from uniformity (χ^2^ test = 2909.852, *p* < 0.001).

Based on the results of this study, seasonal variations in pathogen circulation were observed throughout the year. The first trimester showed the highest proportion of positive results for several pathogens: influenza A virus (94.2%, 1436 of 1524 Influenza A cases in this study), respiratory syncytial virus (86.5%, 668/772), *Bordetella pertussis* (75.9%, 371/489), and adenovirus (42.1%, 152/361).

In the first half of the year, the highest number of *Mycoplasma pneumoniae* detections occurred, with 33.7% (175/519) of positive results recorded in the first trimester and 37.2% (193/519) in the second trimester. Similarly, the influenza B virus was most frequently detected in the first trimester (44.7%; 63/141), followed by the second trimester (31.2%; 44/141). Human metapneumovirus showed a comparable pattern, with 57.1% (250/438) of cases in the first trimester and 36.8% (161/438) in the second trimester.

In contrast, some pathogens were detected throughout the year. Parainfluenza virus was detected year-round, with the highest proportion of positive results in the fourth trimester (34.8%; 288/828), followed by the third trimester (26.8%; 222/828) and the first trimester (26.2%; 217/828). Rhinovirus/enterovirus was also detected throughout the year but was most prevalent during the colder seasons, with 34.4% (1413/4104) of positive results in the fourth trimester and 31.7% (1302/4104) in the first trimester.

The fourth trimester showed the highest proportion of positive *Chlamydia pneumoniae* detections (54.5%; 193/354), followed by the third trimester (37.9%; 134/354).

The highest number of positive SARS-CoV-2 detections was observed during the summer months (third trimester, July–September), accounting for 48.1% (483/1004) of all positive cases, while 30.6% (307/1004) were detected in the first trimester.

## 4. Discussion

In this study, we present the results of all panels tested for respiratory pathogens using a multiplex PCR method at the Teaching Institute for Public Health of Split-Dalmatia County between 1 January and 31 December 2024. A total of 15,437 panels were analyzed, and 8878 (57.5%) were positive. Because some panels contained multiple positive pathogens, 10,546 individual positive results were recorded.

Although nearly 10% more panels were tested for females than for males, the proportion of positive results was significantly higher in males (55.5% among tested females versus 59.9% among tested males). This suggests that female patients may be more likely to seek medical care earlier or more frequently, whereas male patients may be more inclined to present only when symptoms become more pronounced. Greneveld et al. reported a similar pattern: although more female patients were tested, male patients were more frequently referred for additional diagnostic procedures [23]. This sex difference was most evident among individuals older than 19 years, who decide independently whether to seek testing. Among children and adolescents, parental decision-making likely reduces sex-based differences.

Despite all being referred under a clinical diagnosis of respiratory infection, 42.5% of the tested panels were negative. A considerable proportion, approximately 40%, has been reported by other authors [3]. Negative panels may reflect infections caused by pathogens not included in the panel or non-infectious conditions such as allergies, for which antibiotic therapy is not warranted. In such cases, multiplex panels are valuable for confirming or excluding an infectious etiology. By identifying the causative pathogen, multiplex PCR supports more accurate prognostic assessment, more appropriate therapeutic decision-making, and ultimately improves patient outcomes.

High positivity rates were observed among infants, preschool-aged children, and school-aged children (82.5%, 80.0%, and 62.6%, respectively), which is expected given their still-developing immune system and frequent close contact in kindergartens and school settings that facilitate prolonged indoor exposure and efficient respiratory pathogen transmission. As immunity matures with age, the frequency of infections decreases; accordingly, a lower proportion of positive cases was recorded in older age groups (43.4–55.7%), which is consistent with findings elsewhere [2].

Rhinovirus/enterovirus was the most frequently detected pathogen (38.9%), among all age groups except older adults, which is consistent with findings of other authors [2,24], followed by influenza A virus (14.5%) and SARS-CoV-2 (9.5%). Notably, two of the three most common pathogens (influenza A virus and SARS-CoV-2) are vaccine-preventable, although vaccination status was not collected in this study.

Although rhinovirus infections are often clinically mild, their high frequency translates into a substantial public health and economic burden. Rhinoviruses are the most common cause of upper respiratory tract infections and account for a large proportion of medically attended respiratory illnesses, often resulting in repeated outpatient visits, school and work absenteeism, and associated indirect costs, with estimates suggesting that rhinovirus-related illnesses contribute to high costs in healthcare utilization and productivity loss annually [25]. Importantly, rhinovirus is also a major trigger of asthma exacerbations and wheezing illnesses in children, contributing to increased morbidity, emergency department use, and hospitalizations [26]. Thus, the high rate of rhinovirus/enterovirus detections in our study reflects not only frequent community circulation but also a meaningful clinical and socioeconomic impact that extends beyond simple upper respiratory symptoms.

In our study, both adults and children demonstrated high viral infection rates, but specific pathogen distributions varied by age groups. Rhinovirus/enterovirus was the most frequently detected pathogen across nearly all age groups.

Viruses dominated among infants (<1 year) and preschool children aged 1–6 years. Therefore, viral infection should be the primary diagnostic consideration in these age groups. In addition to rhinovirus/enterovirus, influenza A virus, respiratory syncytial virus, and parainfluenza viruses were frequently detected among preschool and school-aged children, which is consistent with established epidemiological patterns indicating that children are primary drivers of influenza transmission and seasonal epidemics [27]. Although influenza can be prevented by vaccination, healthy children are not routinely included among the recommended risk groups for vaccination in Croatia [28]. Regarding RSV, in Croatia, RSV prevention currently relies on palivizumab, a recombinant humanized monoclonal antibody used for seasonal immunoprophylaxis in preterm infants and selected high-risk patients, according to national recommendations [29]. At present, long-acting monoclonal antibodies (e.g., nirsevimab, clesrovimab) and maternal RSV vaccination are not routinely available. While newer preventive approaches have been shown to reduce RSV-related hospitalizations and severe disease in infants, their impact on overall community circulation of RSV is likely to be limited, as passive immunization primarily reduces disease severity rather than transmission [10,11].

In addition to the rhinovirus/enterovirus and influenza A virus, bacterial pathogens such as *Mycoplasma pneumoniae*, *Bordetella pertussis*, and *Chlamydia pneumoniae* were prevalent among school children aged 7–14 years. These pathogens should be considered in differential diagnosis, and antibiotic therapy is warranted when bacterial infection is confirmed. A similar pattern was observed among adolescents.

Among adults aged 19–64 years, viruses dominated—mainly rhinovirus/enterovirus, influenza A, and SARS-CoV-2. Among older adults aged ≥65 years, viruses—primarily SARS-CoV-2, followed by influenza A virus—were the most frequently detected pathogens.

Although *Bordetella pertussis* infections peaked in adolescents aged 7–14 years, a substantial proportion of cases were also recorded in adults. In adolescents and adults, pertussis often presents atypically, with a less pronounced or absent characteristic cough, increasing the likelihood of missed diagnoses and continued transmission to vulnerable individuals, especially unvaccinated infants and pregnant women in late gestation [30]. Pertussis is a vaccine-preventable disease and is included in the Croatian National Immunization Programme [28]; however, vaccination status was not available for this study.

Co-infections were common, accounting for almost one-fifth (17.4%) of all positive panels (16.0% double and 1.4% triple infections), mostly involving combinations of respiratory viruses. Wide variation in reported co-infection in the literature likely reflects differences in diagnostic methodology, panel composition, and study design [2,3]. Rhinovirus/enterovirus was the most common component of co-infections, especially when combined with parainfluenza viruses, while *Bordetella pertussis* was the most common bacterial co-pathogen. The clinical relevance of co-infections, particularly whether they increase disease severity, remains an important question. Viral interactions can be synergistic, enhancing viral replication and exacerbating disease, or antagonistic, with one virus inhibiting the replication of the other or modulating certain aspects of the immune response. Understanding the mechanisms behind this variety of synergies and antagonisms of viral co-infections remains mostly unclear [31]. However, several studies have shown that respiratory viral co-infections can worsen clinical severity and lead to poorer outcomes [32,33,34].

Seasonal analysis, in our study, showed that nearly half (47.0%) of all positive results occurred in the first quarter of the year, followed by the fourth quarter (22.8%), reflecting typical winter seasonality.

In the first trimester, there was the highest proportion of positive results for several pathogens: influenza A virus, respiratory syncytial virus, *Bordetella pertussis*, and adenovirus, while the highest proportions of *Mycoplasma pneumoniae*, influenza B virus, and human metapneumovirus occurred in the first half of the year (the first and second trimesters), which is partially consistent with the findings of Tang et al., who reported that *Mycoplasma pneumoniae* infections peak during the summer [24].

Rhinovirus/enterovirus and parainfluenza virus circulated throughout the year, though more commonly during the colder months, with peaks in the fourth and first trimesters, which is partially consistent with the findings of Berginc et al.; however, unlike their reported spring peak for rhinovirus, the second peak in our study occurred in winter [35].

More than half of the positive *Chlamydia pneumoniae* results were detected in the fourth trimester, with a peak among school-aged children beginning in October, coinciding with the start of the school year, which is consistent with the results of Chen et al. [36].

SARS-CoV-2 circulation was highest during the third trimester (July–September, in 48.1% cases). Studies on the seasonality of COVID-19 have yielded inconsistent conclusions: Wiekman et al. observed a peak from November to April [37], whereas Shamsa et al. reported a summer increase, aligning with our findings [38]. In 2024, with no mandatory testing for travelers, several behavioral factors likely contributed: increased time spent in air-conditioned indoor spaces, reduced ventilation due to the closed windows, and a high level of travel and tourism, including travelers from regions with more cases of COVID-19. Mild symptoms, such as fatigue, headache, or sore throat, may also have been attributed to travel-related discomfort rather than infection, facilitating unnoticed transmission. A second peak in the first trimester (30.6%) reflected the year-round circulation and rapid evolution of SARS-CoV-2. Throughout 2024, the Omicron variant and its highly mutated sub-variants predominated [39].

Currently, in our setting, the cost of a multiplex PCR test with prior automated nucleic acid extraction is approximately €25 per sample. Rapid antigen point-of-care diagnostic tests are considerably less expensive (approximately €5 per test), but they have several limitations: sensitivity may be lower, multiple individual tests are often required to cover relevant pathogens, and some pathogens are not included at all. Importantly, we consider rapid testing to be most effective when performed directly in primary care settings rather than in a microbiological laboratory. However, implementing point-of-care testing in general practice would increase costs and operational requirements for primary care providers, which may limit its adoption. This contributes to the continued empirical prescribing of antibiotics for respiratory infections, underscoring the clinical and public health relevance of our study.

However, we do not consider routine multiplex PCR testing for all patients with respiratory infections to be cost-effective. The differentiation between viral and bacterial infections relies not only on diagnostic testing but also on clinical interpretation in the context of the patient’s presentation. Our findings confirm that respiratory infections are predominantly viral, underscoring the value of microbiological testing in guiding antibiotic therapy when clinical assessment alone cannot reliably distinguish between viral and bacterial infections, reducing unnecessary antibiotic use, and limiting the development of antimicrobial resistance. Distinct age-related patterns were observed: viral pathogens predominate in preschool-aged children, influenza viruses occur across all age groups, SARS-CoV-2 is more common in older patients, and bacterial infections are more frequent in school-aged children. These findings support a targeted testing approach based on age, clinical severity, and the likely causative pathogens. The cost-effectiveness of PCR testing should also be considered in the context of reducing unnecessary antibiotic use and its long-term societal costs, including antimicrobial resistance, which remains a major global public health problem [40]. When antibiotic therapy is being considered for a respiratory infection, multiplex PCR testing to confirm a bacterial etiology before treatment initiation may be more beneficial than empirical antibiotic use when a viral infection is possible.

Therefore, although our results cannot directly imply immediate changes to the mandatory or recommended national immunization program, they provide important evidence to inform future vaccination planning and policy discussions. Based on the observed age-distribution peaks, several targeted public health implications can be highlighted. The pronounced burden of influenza among preschool and school-aged children may contribute to future discussions on vaccination strategies, such as expanding influenza vaccination recommendations beyond currently defined risk groups in line with practices adopted in several European countries [41,42]. Such an approach could reduce transmission in the community and indirectly protect vulnerable populations. Currently, the target groups for influenza vaccination include individuals aged ≥ 65 years, people with chronic disease, healthcare workers, residents and staff working in long-term care facilities, and pregnant women [29]. Regarding pertussis, our findings are consistent with recent national policy changes implemented in 2025 in response to the pertussis epidemic, including the introduction of vaccination recommendations for pregnant women from the 16th week of pregnancy [29]. Our data further support the continuation of this strategy, given the observed age-related disease burden and the importance of protecting young infants.

### 4.1. Clinical and Public Health Implications

Our findings move beyond mere epidemiological reporting and offer three actionable insights for regional healthcare.

With an 87.0% viral predominance observed in our 15,437-sample cohort, our data provide a quantitative justification for clinicians to withhold empirical antibiotics in outpatient settings, potentially preventing thousands of unnecessary prescriptions annually in Split-Dalmatia County.

The discovery of a dominant SARS-CoV-2 summer peak (48.1%) and early-year peaks for *B. pertussis* and *M. pneumoniae* suggests that local health authorities must decouple ‘respiratory season’ from ‘winter season.’ This necessitates a year-round allocation of diagnostic resources, particularly during the peak tourism months.

The observed higher positivity rates in children suggest that age-targeted testing strategies may represent a promising approach, but this concept requires validation in prospective and cost-effectiveness studies.

Our findings provide hypothesis-generating insights that may inform future research and potential optimization of diagnostic strategies. However, given the descriptive nature of this study, further analyses are required before translating these observations into clinical or policy recommendations.

### 4.2. Strengths and Limitations of the Study

This study is limited to individuals tested at a single outpatient laboratory and excludes those tested at a hospital laboratory, individuals who used home tests, those diagnosed clinically without laboratory confirmation, and those who did not seek medical care. Consequently, the results do not represent the true population-level burden. Our findings reflect an outpatient population in a single-center setting. Therefore, caution should be exercised when generalizing them to hospital settings or broader populations. However, because this is the largest laboratory serving the entire county, the findings likely approximate real circulation patterns.

A further significant limitation is the absence of granular data on clinical symptoms and longitudinal outcomes. This lack of clinical correlation precludes a definitive assessment of the real-world impact of multiplex PCR testing on patient management and the specific role of co-infections in driving disease severity. Since our study exclusively enrolled outpatients—who are mostly present with milder clinical manifestations—the results may not reflect the pathogen dynamics or complication rates seen in hospitalized populations. Furthermore, the absence of detailed demographic and clinical profiles prevented the identification of high-risk individuals or those with underlying comorbidities. Consequently, our findings regarding co-infection complexity and seasonal shifts should be interpreted as epidemiological trends that require further validation through clinical and laboratory studies. The absence of vaccination data restricts our ability to analyze potential correlations between immunization levels and the shifting trends in respiratory pathogen detection during the post-pandemic period.

A major strength of this study is its large sample size, encompassing the entire population of Split-Dalmatia County, one of Croatia’s largest counties. Year-round surveillance allowed detailed evaluation of circulation patterns for the 13 most common respiratory pathogens.

## 5. Conclusions

This study provides the first comprehensive evidence of a reconfigured post-pandemic respiratory landscape in the Croatian Mediterranean. Our data reveal that traditional seasonal boundaries have blurred, with significant off-season circulation of both viral and bacterial pathogens.

The practical impact of this work is twofold: first, it establishes multiplex PCR as an essential tool for antibiotic stewardship, proving that nearly 9 out of 10 outpatient infections do not require antibacterial treatment. Second, it identifies specific high-risk age groups and temporal peaks that should guide future regional vaccination and testing policies. These findings serve as a strategic roadmap for clinicians and public health professionals to transition from empirical, one-size-fits-all treatments to a data-driven, precision diagnostic approach.

## Figures and Tables

**Table 1:** Distribution of tested panels by age group and sex (N = 15,437).

Age Group (Year)		Study Population		Total Population in Split-Dalmatia County (%) *
	Females	Males	Total (%)	
<1	93	113	206 (1.3)	4031 (1.0)
1–6	1599	1788	3387 (21.9)	5581 (5.6)
7–14	1270	1500	2770 (17.9)	8255 (8.2)
15–18	556	597	1153 (7.5)	17,783 (4.2)
19–64	4042	2309	6351 (41.1)	250,895 (59.2)
≤65	923	647	1570 (10.2)	92,110 (21.8)
Total	8483 (55.0%)	6954 (45.0%)	15,437 (100.0%)	423,407 (100.0)

* The Census of Population, Households and Dwellings in the Republic of Croatia. Available at: https://dzs.gov.hr/u-fokusu/popis-2021/popisni-upitnik/english/1361 (accessed on 14 January 2026).

**Table 2:** Distribution of positive respiratory test panel results by age group and pathogen (N = 10,546).

Pathogen	Age Groups (Years)						
	<1	1–6	7–14	15–18	19–64	≤65	Total
	Infants	Preschool Children	School-Aged Children	Adolescents	Adults	Older Adults	
Rhinovirus/enterovirus	129	1718	772	244	1039	202	4104
Influenza A virus	1	229	302	168	710	114	1524
SARS-CoV-2	22	126	87	38	518	213	1004
Human parainfluenza virus	34	426	103	28	179	58	828
Respiratory syncytial virus	10	396	132	46	134	54	772
*Mycoplasma pneumoniae*	2	95	240	69	107	6	519
*Bordetella pertussis*	4	112	157	45	146	25	489
Human metapneumovirus	5	168	92	33	99	41	438
Adenovirus	6	248	49	15	42	1	361
*Chlamydia pneumoniae*	4	83	139	23	104	1	354
Influenza B virus	0	30	36	17	56	2	141
*Bordetella parapertussis*	0	3	3	0	3	2	11
*Legionella pneumophila*	0	1	0	0	0	0	1
Total	217	3635	2112	726	3137	719	10,546

## Data Availability

The raw data supporting the conclusions of this article will be made available by the authors on request.

## Abbreviations

The following abbreviation is used in this manuscript:

TIPH SDC	Teaching Institute for Public Health of Split-Dalmatia County
